# Vacuum-Compression Therapy as an Adjunct to Physical Therapy in Patients with Knee Osteoarthritis: A Pilot Comparative Study

**DOI:** 10.3390/bioengineering13050563

**Published:** 2026-05-16

**Authors:** Diana-Lidia Tache-Codreanu, Ana-Maria Tache-Codreanu, Lucian Bobocea, Teodor Dan Poteca, Andrei Tache-Codreanu, Cosmin-Alec Moldovan, Corina Sporea

**Affiliations:** 1 Department of Medical, Surgical and Preventive Disciplines, Faculty of Medicine, Titu Maiorescu University, 031593 Bucharest, Romania; dianatache@yahoo.com (D.-L.T.-C.); cosmin.moldovan@prof.utm.ro (C.-A.M.); 2 Medical Rehabilitation Department, Colentina Clinical Hospital, 020125 Bucharest, Romania; bobocea.lucian95@gmail.com; 3 Faculty of Medicine and Farmacy, University of Medicine and Pharmacy “Carol Davila”, 020021 Bucharest, Romania; ana-maria.tache2024@stud.umfcd.ro; 4 Surgical Department, Colentina Clinical Hospital, 020125 Bucharest, Romania; 5 Faculty of Theatre, National University of Theatre and Film “I.L. Caragiale”, 021452 Bucharest, Romania; 6 Department of General Surgery, Witting Clinical Hospital, 010243 Bucharest, Romania; 7 Discipline of Balneophysiokinetotherapy and Recovery, Faculty of Midwifery and Nursing, University of Medicine and Pharmacy “Carol Davila”, 020021 Bucharest, Romania; corina.sporea@gmail.com; 8 Scientific Research Core, National University Center for Children’s Neurorehabilitation “Robanescu-Padure”, 041408 Bucharest, Romania

**Keywords:** knee osteoarthritis, physical therapy, vacuum-compression therapy, rehabilitation, functional outcomes

## Abstract

Background: Knee osteoarthritis (OA) is one of the leading causes of disability in older adults. As definitive treatment often involves knee replacement surgery, effective non-invasive approaches capable of alleviating symptoms and preserving mobility are needed to delay surgical intervention or bridge waiting periods for surgery. Methods: Thirty-two patients with knee OA were included in this pilot comparative study. Patients underwent either a standardized physical therapy program (10 sessions) or the same program supplemented with vacuum-compression therapy (VCT), according to treatment received during routine clinical care. Outcome measures included the Western Ontario and McMaster Universities Osteoarthritis Index (WOMAC), Visual Analog Scale (VAS), and the Physical and Mental Component Summary scores of the SF-12 questionnaire (PCS, MCS). Assessments were performed at baseline and at 1-month follow-up, with WOMAC additionally evaluated immediately after treatment. Responder analysis based on minimal clinically important difference (MCID) thresholds was also performed. Results: Both groups demonstrated significant improvement across most outcomes. Between-group analysis showed greater improvements in the intervention group, with statistically significant differences observed for functional outcomes (WOMAC and PCS). Conclusions: In this pilot comparative study, the addition of VCT to standard physical therapy was associated with greater functional improvement in patients with knee OA.

## 1. Introduction

Knee osteoarthritis (OA) is a frequently occurring, progressive, and multifactorial degenerative joint disease, representing one of the most prevalent forms of osteoarthritis worldwide [1,2]. The prevalence of symptomatic knee OA reaches approximately 13% among women over 60 years of age and around 10% among men in the same age group, with up to one quarter of affected individuals experiencing severe functional limitation [3,4,5]. Epidemiological data consistently indicate a greater impact in women, in whom knee OA is among the leading contributors to disability compared to men [6]. The overall health burden associated with knee OA is substantial and has been reported to be comparable to that of other major chronic conditions in older adults [4]. This significant functional impairment translates into increased healthcare utilization and economic burden [2,7]. Health economic analyses comparing patients with knee OA to matched controls have demonstrated markedly higher all-cause healthcare costs in affected individuals, in some reports nearly double those observed in matched controls [8].

At early stages, knee OA is predominantly characterized by activity-related pain associated with repetitive loading or joint overuse. As the condition progresses to moderate and advanced stages, patients increasingly experience joint instability, muscle weakness, and functional impairment, often accompanied by pain occurring with minimal exertion or even at rest, including nocturnal pain [9]. It has been well documented that pain, restricted mobility, and limitations in function and gait substantially impair quality of life among patients with knee OA [10,11,12,13]. This deterioration becomes more pronounced in advanced stages of the disease, particularly during the often prolonged waiting period for total knee arthroplasty (TKA) in many healthcare systems [14,15,16]. Although TKA is considered the standard definitive intervention for advanced knee OA after failure of nonoperative management, patients frequently endure months or even years of markedly reduced mobility and diminished quality of life while awaiting surgery [1,17,18,19]. Therefore, identifying additional therapeutic strategies capable of alleviating symptoms, preserving functional mobility, and potentially delaying surgical intervention or bridging the preoperative waiting period remains of considerable clinical importance [2,7,15,16].

Initial management of knee OA typically includes patient education emphasizing weight management and avoidance of excessive joint loading, along with structured exercise therapy aimed at improving physical function and addressing muscle weakness. Physiotherapeutic interventions are commonly employed to enhance local circulation, reduce inflammation, and alleviate pain [20,21,22,23,24,25,26]. In addition, various pharmacological approaches and oral supplements are frequently used to provide short-term symptom relief [9,,27]. While exercise therapy is generally considered a first-line treatment in knee OA, the evidence supporting many physical therapy modalities and adjunctive approaches remains inconsistent [28,29,30,31]. Reported effects are often heterogeneous, with considerable variability in patient response across studies [24,32,33,34,35,36].

Over the past decades, vacuum-compression therapy (VCT) has been proposed as a potentially promising modality for improving lower-limb perfusion and tissue condition. Its mechanism of action is based on the cyclic alternation between phases of positive pressure (compression) and negative pressure (suction). During treatment, the lower limb is typically placed within a sealed chamber connected to a pressure-generating system, allowing controlled modulation of external pressure. The compression phase facilitates venous and lymphatic drainage, whereas the suction phase may facilitate arterial inflow. This dynamic pressure modulation may enhance tissue oxygenation and microvascular perfusion, thereby supporting local metabolic processes [37,38,39,40]. From a pathophysiological perspective, improved joint perfusion and circulation may influence inflammatory processes within the knee joint, including synovitis, and contribute to symptom modulation and functional improvement in knee OA [41,42,43,44,45].

To date, evidence regarding the use of vacuum-based therapies in knee OA remains limited, with only a single study investigating the effects of a similar technology in this population. Ionescu et al. reported statistically significant improvements following intermittent vacuum therapy; however, no statistically significant differences between groups were identified when compared with conventional treatment modalities, which were also administered in the intervention group [46]. Given the distinct biomechanical and circulatory effects associated with the combined application of compression and suction, therapeutic responses may differ when both pressure modalities are integrated within a single intervention protocol. Importantly, to the best of our knowledge, no study has systematically evaluated the clinical effects of combined compression–suction therapy in patients with knee OA.

The aim of the present pilot comparative study was to assess the feasibility and potential clinical benefit of integrating VCT into standard physical therapy and to examine its impact on clinical and functional outcomes between groups.

## 2. Materials and Methods

### 2.1. Study Design

This investigation was carried out at a single center as a parallel-group pilot comparative study within routine clinical practice at the Department of Medical Rehabilitation, Colentina Clinical Hospital, Bucharest, Romania, between October 2025 and January 2026. The study compared two cohorts of patients with knee osteoarthritis treated within routine clinical care: those who received standard rehabilitation alone and those who received rehabilitation supplemented with VCT, with data extracted retrospectively from clinical records.

Given the non-randomized design, treatment allocation was not controlled by the investigators but reflected routine clinical decision-making and patient preference. Patients were informed about the treatment procedures, potential risks, and expected benefits of the intervention at the time of care and provided written informed consent.

The study adhered to the principles of the Declaration of Helsinki and received approval from the Research Ethics Committee of Colentina Clinical Hospital, Bucharest (approval number 23, approved on 24 July 2025).

### 2.2. Participants

Patients with symptomatic knee OA who underwent a rehabilitation program at the Department of Medical Rehabilitation, Colentina Clinical Hospital, Bucharest, Romania, during the study period were identified and included in the analysis. Diagnosis was established based on clinical examination and radiographic findings consistent with Kellgren–Lawrence (KL) grade 1–3. To be included, participants had to be at least 18 years of age and able to participate in the prescribed physical therapy program. Bilateral knee OA was permitted. OA affecting other joints was allowed if present in a stable and non-progressive stage. Inclusion required a minimum interval of one month after intra-articular knee injection and six months after knee arthroscopy.

As part of routine clinical care, patients were advised to avoid additional therapeutic interventions, including intra-articular infiltrations, and not to initiate new pharmacological treatments beyond the prescribed rehabilitation protocol. They were also advised to keep their habitual level of physical activity and lifestyle habits without substantial changes. Any relevant medical changes or need for additional treatment were monitored and recorded in the clinical documentation.

Patients were excluded if they presented with acute venous thrombosis or embolism (or suspected thromboembolic disease), aneurysm in the treated limb, severe bleeding disorders or high-risk anticoagulant therapy, acute open wounds with risk of bleeding or subcutaneous emphysema, local purulent infection or advanced necrosis, local malignant tumors, limb edema of cardiac, nephrogenic, or hepatogenic origin, decompensated heart failure associated with edema, septic conditions, hyperthermia, pregnancy, severe psychiatric disorders preventing safe participation, or other contraindications to VCT. Relative contraindications, including hypertension, atherosclerosis, ischemic heart disease, orthostatic hypotension, or a tendency toward phlebothrombosis, were individually assessed by the treating physician and patients were excluded if these conditions were present in severe or decompensated stages.

### 2.3. Study Groups

Patients were assigned to treatment groups according to the rehabilitation protocol received within routine clinical practice. Treatment selection was based on clinical indication and patient preference following explanation of the therapeutic procedures, potential benefits, and contraindications. Patients who received standard rehabilitation alone constituted the control group, while those who underwent rehabilitation supplemented with VCT formed the intervention group.

Standard physical therapy sessions were delivered by physical therapists according to the routine rehabilitation practices of the department. Outcome measures were collected by physical therapists using standardized assessment procedures. VCT was administered by a therapist trained and experienced in the use of the VCT device.

### 2.4. Interventions

All participants underwent a standardized rehabilitation program delivered twice weekly for a total of 10 sessions. Each session included therapeutic exercise focused on improving joint mobility and muscle strength, joint mobilization techniques, and strengthening exercises targeting periarticular musculature. Adjunctive physical therapy modalities were applied, including low-frequency analgesic electrical stimulation, deep thermotherapy using shortwave diathermy, and muscle-relaxation massage techniques. The control group received this rehabilitation protocol alone.

Participants in the intervention group received VCT using a vacuum-compression device (BTL Industries Ltd., Prague, Czech Republic) in addition to the standard rehabilitation program. During each session, the patient was seated comfortably in the therapy chair, and the treated lower limb was fitted with an appropriately sized cuff and sealing components selected according to individual anatomical characteristics. The limb was then positioned within the VCT chamber to ensure proper sealing and safe pressure modulation.

A standardized cyclic pressure protocol was applied for 30 min per session, twice weekly over 10 sessions. The intervention consisted of alternating phases of positive pressure set at +6 kPa for 45 s and negative pressure set at −4 kPa for 10 s. The protocol was designed to enhance lower-limb perfusion, improve microcirculation, and potentially modulate inflammatory processes. Throughout the treatment, the therapist monitored skin coloration and patient tolerance via the transparent chamber. Minor adjustments to pressure intensity or phase duration were permitted, when necessary, based on tissue response or patient feedback, while remaining within predefined therapeutic limits. No sham or placebo-controlled intervention was applied to the control group.

### 2.5. Outcome Measures

Outcome measures were evaluated at study entry and again one month after completion of the intervention. In addition, the WOMAC score was evaluated immediately after completion of the intervention period. All questionnaires were completed independently by participants.

The main outcome of interest was functional status assessed using the Western Ontario and McMaster Universities Osteoarthritis Index (WOMAC), Likert version. The WOMAC evaluates three domains: pain, stiffness, and physical function. Scores were normalized to a 0–100 scale, where higher scores reflect greater symptom severity and functional limitation. A score of 100 reflects extreme difficulty in performing daily activities, whereas a score of 0 indicates no functional impairment [47].

Pain intensity, assessed using the Visual Analog Scale (VAS), was included as a secondary outcome and health-related quality of life was captured using the 12-Item Short Form Health Survey (SF-12). The VAS was rated on a 0–10 scale, where 0 indicates no pain and 10 represents the highest pain intensity imaginable [48]. The SF-12 provides two summary measures: the Physical Component Summary (PCS) and the Mental Component Summary (MCS), where higher values reflect better self-perceived health status [49].

For responder analysis, minimal clinically important difference (MCID) thresholds were predefined as follows: ≥12-point improvement for WOMAC, ≥2-point reduction for VAS, and ≥5-point improvement for both PCS and MCS [50,51,52].

### 2.6. Sample Size

This study was designed as a pilot comparative study primarily aimed at evaluating feasibility and estimating preliminary effect sizes. For this reason, no prospective sample size estimation was conducted, and the cohort size was driven by the pool of eligible patients available during the study period and was considered sufficient for exploratory analysis and hypothesis generation for future studies.

### 2.7. Statistical Analysis

All analyses were carried out using R (version 4.5.2; R Foundation for Statistical Computing, Vienna, Austria). Normality of continuous variables was examined with the Shapiro–Wilk test. As normality assumptions were not met for several variables, descriptive statistics were presented as median and interquartile range (IQR).

Within-group changes from baseline to 1-month follow-up were evaluated using the Wilcoxon signed-rank test. Between-group comparisons at 1-month follow-up were conducted using analysis of covariance (ANCOVA), with follow-up scores as the dependent variable, treatment group as the independent variable, and baseline values included as covariates. Given the non-randomized design, ANCOVA was used to partially adjust for baseline differences between groups. Adjusted mean differences with 95% confidence intervals (CI) were reported. Effect sizes were expressed as partial eta squared (η^2^), calculated using Type III sums of squares.

For WOMAC, an additional longitudinal analysis was conducted using a linear mixed-effects model that included baseline, post-treatment, and 1-month follow-up assessments, with group, time, and their interaction specified as fixed effects and participant as a random intercept.

Responder analysis was conducted based on predefined MCID thresholds. Between-group differences in responder proportions were quantified using risk difference and risk ratio with corresponding 95% confidence intervals. All analyses were performed on a complete-case basis. A significance level of *p* < 0.05 was used.

## 3. Results

**Figure 1:**
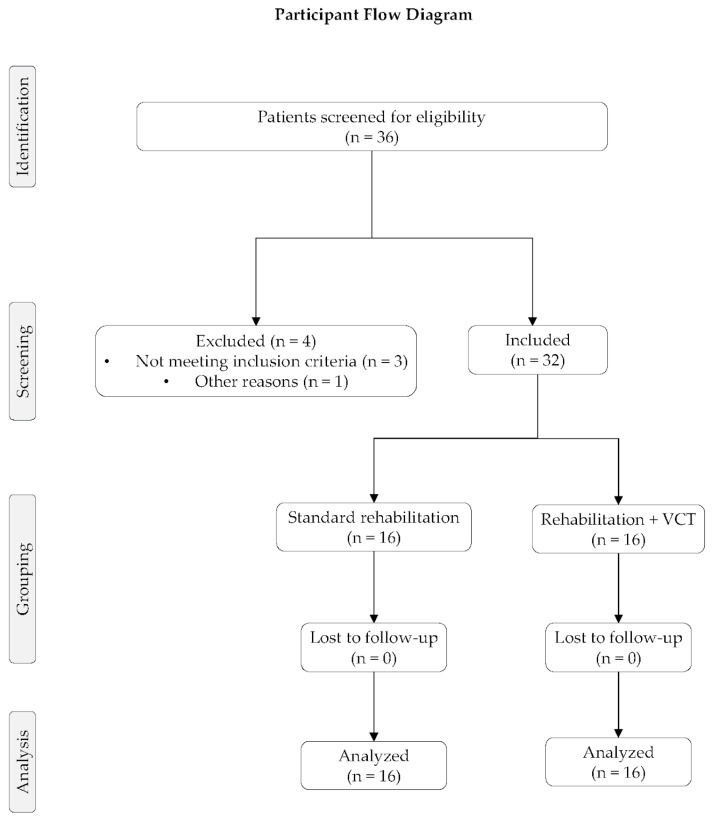
Flow diagram of patient selection, grouping, follow-up, and analysis.

Of the 36 patients screened for eligibility, 32 were included in the analysis (16 in the control group and 16 in the intervention group). All included participants completed the intervention protocol and follow-up evaluations, allowing inclusion of all participants in the final analysis. No adverse events were reported during the study period. The flow of participants throughout the study is presented in Figure 1. Baseline characteristics of both groups are summarized in Table 1.

**Figure 2:**
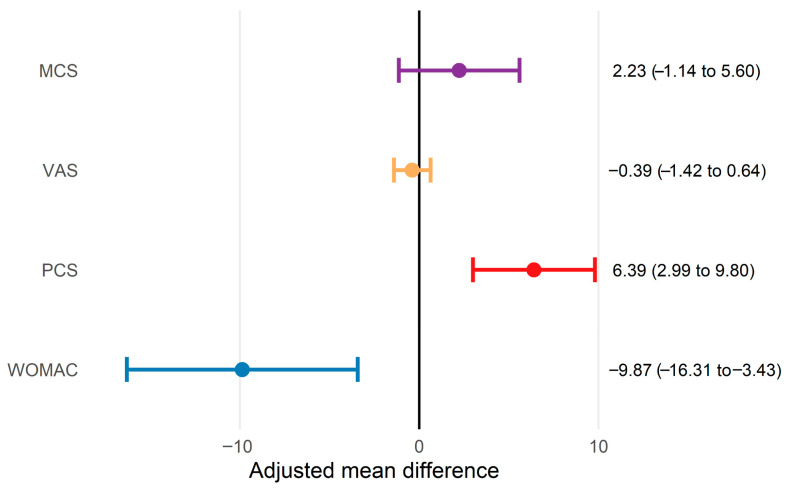
Baseline-adjusted between-group differences (intervention minus control) at 1-month follow-up (ANCOVA). Points represent adjusted mean differences, and lines denote 95% confidence intervals.

Baseline characteristics were generally comparable between groups, although a higher proportion of female participants and slightly higher BMI values were observed in the intervention group. Both groups demonstrated statistically significant within-group improvements across all outcome measures at 1-month follow-up, with larger median percentage changes observed in the intervention group (Table 2). Between-group comparison adjusted for baseline values indicated greater improvement in the intervention group for functional outcomes assessed by WOMAC and SF-12 PCS (Table 3). Both outcomes demonstrated large effect sizes, and their 95% confidence intervals did not cross zero, indicating a between-group effect (Figure 2). However, no statistically significant between-group differences were observed for pain intensity (VAS) or mental health (MCS). In the ANCOVA models, baseline values were significant predictors of follow-up scores, particularly for WOMAC and PCS, supporting the use of baseline adjustment in this non-randomized design.

**Figure 3:**
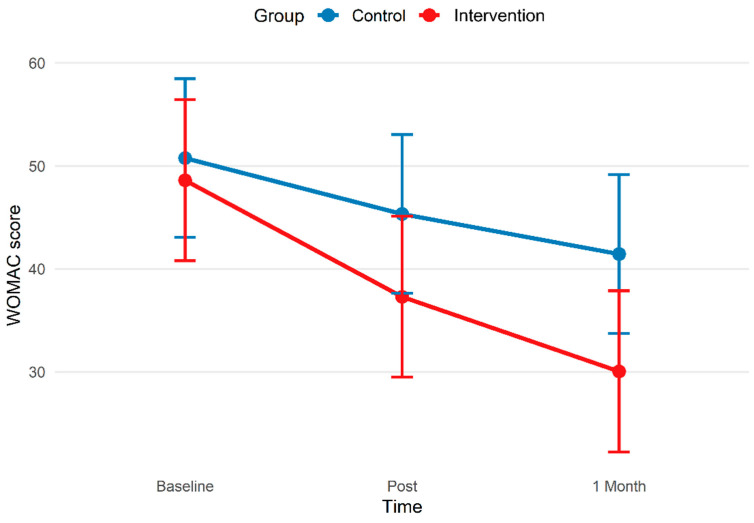
Changes in WOMAC total scores over time in the control and intervention groups. Estimated means are displayed as points, and the associated error bars illustrate 95% confidence intervals.

In addition to the primary ANCOVA analysis at 1-month follow-up, WOMAC scores were further analyzed using a linear mixed-effects model including all three time points (baseline, post-treatment, and 1-month follow-up). The model demonstrated a significant effect of time and a significant main effect of group. However, the group × time interaction was not statistically significant, indicating a comparable trajectory of improvement in both groups (Figure 3).

**Figure 4:**
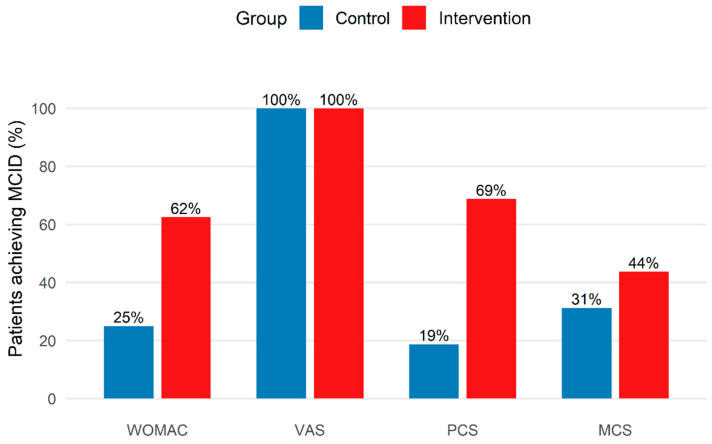
Percentage of participants achieving predefined minimal clinically important difference (MCID) thresholds at 1-month follow-up in the control and intervention groups.

The MCID responder analysis at 1-month follow-up was consistent with the between-group differences observed for functional outcomes (WOMAC and PCS), as shown in Table 4 and Figure 4. The absolute difference in responder rates suggests that a higher proportion of participants in the intervention group achieved clinically meaningful improvement compared with controls. In relative terms, participants receiving the combined intervention were more likely to achieve clinically relevant improvement in WOMAC and PCS. In contrast, all participants met the predefined MCID threshold for pain (VAS), precluding detection of a between-group difference. Similarly, clinically meaningful changes in mental health (MCS) were distributed comparably between groups.

## 4. Discussion

The present pilot comparative study suggests that the addition of VCT to standard physical therapy may be associated with greater functional improvement in patients with knee OA. Compared with rehabilitation alone, the combined intervention was associated with greater gains in functional status, as reflected by WOMAC and the physical component of the SF-12. These findings were consistent across both baseline-adjusted analyses and clinically meaningful responder outcomes.

In contrast, pain intensity did not differ significantly between the groups. Notably, all participants in both groups achieved the predefined MCID threshold for VAS, suggesting a potential ceiling effect that may have limited the ability to detect additional between-group differences despite numerically greater percentage improvement in the intervention group [24,34].

Similarly, clinically meaningful improvement in the mental component of the SF-12 was less pronounced than for functional outcomes and was distributed comparably between groups. This finding suggests that short-term functional gains may not immediately translate into measurable changes in perceived mental health, particularly within a short follow-up period, compared with other categories of physical modalities [2,36,53,54,55,56].

Overall, these findings indicate that the potential benefit of VCT may lie primarily in enhancing physical function, which represents a key determinant of independence and quality of life in patients with knee OA [23,24,25].

The observed between-group difference in functional improvement may reflect, at least in part, the targeting of different tissues and mechanisms by standard rehabilitation and VCT. While standard rehabilitation primarily targeted pain reduction and improvement of physical function through muscle strengthening, aerobic, and neuromuscular exercise, VCT may act through dynamic pressure modulation, influencing tissue oxygenation, microvascular perfusion, and local metabolic exchange [23,24,25,26,38,40,44,45,57,58]. Impaired lymphatic drainage and reduced clearance capacity have been described in osteoarthritic joints and may contribute to persistent synovial inflammation and functional limitation [41,42,44,45,59]. By enhancing local perfusion and fluid exchange, VCT may contribute to the modulation of the intra-articular environment and support metabolic balance within the joint [42,43]. These mechanisms offer a mechanistically plausible explanation for the more pronounced effects observed in functional outcomes compared with pain intensity alone.

In the broader context of emerging bioengineering strategies for knee OA, recent approaches such as biomaterial-based or regenerative therapies (e.g., hydrogels or nanomaterials) primarily aim to restore cartilage structure or modulate the intra-articular biological environment. These strategies have shown promising results in preclinical and early clinical studies, particularly in terms of tissue regeneration and biochemical modulation [60,61]. In contrast, VCT represents a non-invasive modality targeting the biomechanical and circulatory environment of the joint. Rather than directly modifying tissue structure, VCT may complement these approaches by optimizing local physiological conditions and supporting functional improvement [42,43].

Although existing clinical evidence indirectly supports the role of improved perfusion in modulating inflammation and symptoms in knee OA, direct evidence specifically evaluating the effects of VCT in this population remains limited. To date, the study by Ionescu et al. provides the closest comparison, investigating intermittent vacuum therapy in patients with knee OA [46]. That intervention relied exclusively on negative pressure cycles and did not incorporate alternating compression phases. While statistically significant improvements were observed following the combined application of vacuum therapy and standard rehabilitation, no significant between-group differences were detected compared with standard rehabilitation alone. A key distinction between the present study and that of Ionescu et al. lies in the applied technology, as the incorporation of alternating compression and suction phases in VCT may result in distinct physiological effects compared with negative pressure alone.

The present study has several limitations. First, the non-randomized design and patient self-selection may have introduced selection bias and limited the ability to establish causal relationships. In particular, the formation of groups based on treatment choice rather than random allocation may have resulted in baseline differences between groups that could influence treatment response. Baseline characteristics were generally comparable between groups in terms of disease severity, with a similar distribution of KL grades. However, some differences were observed in BMI distribution, with a higher proportion of normal-weight and obese participants in the intervention group and a higher proportion of overweight participants in the control group. These differences may have influenced treatment response, as body mass index is known to affect joint loading, inflammation, and functional outcomes in knee OA [62]. A higher proportion of female participants was observed in the intervention group, which may have influenced the results, given known sex-related differences in pain perception and functional limitation in knee OA. Previous studies have reported that women with knee OA tend to experience greater pain intensity and functional impairment compared with men, which could have affected the observed treatment response [63,64]. Because of its pilot design and small sample, these results should be viewed as preliminary and may not be representative of the broader knee OA population. Second, the study was conducted at a single center, which may limit external validity for certain outcomes and contributed to wide confidence intervals. Third, follow-up was restricted to one month, limiting the ability to evaluate whether the observed functional improvements are sustained over time or to assess the potential impact of VCT on delaying surgical intervention. Due to the nature of the intervention, neither participants nor treating therapists were blinded, which may introduce performance bias. Furthermore, both groups received standard physical therapy, and the absence of a sham or placebo-controlled intervention limits the ability to distinguish the specific physiological effects of VCT from those of the combined therapeutic approach or from non-specific, expectation-related effects. In addition, no objective physiological or biochemical measurements (e.g., imaging-based perfusion assessments or inflammatory biomarkers) were included, which limits the ability to directly evaluate the underlying mechanisms of action of VCT. Finally, the ceiling effect observed for pain outcomes may have limited the ability to detect additional between-group differences in VAS scores.

Despite its pilot design, the present study indicates a potential functional benefit of VCT in patients with knee OA. The observed effect sizes for functional outcomes provide a preliminary signal that the addition of VCT to standard physical therapy may offer clinically meaningful additional benefits. However, these findings should be considered exploratory and hypothesis-generating rather than definitive evidence of efficacy. From a methodological perspective, the high correlation between baseline and follow-up WOMAC scores (r = 0.77) supports the efficiency of the ANCOVA approach applied in this study. Under conservative assumptions (partial η^2^ ≈ 0.15), approximately 24 participants per group would be required to achieve 80% power in a simple ANOVA framework. Taking into account the strong baseline–follow-up correlation, as well as the potential overestimation of effect sizes in pilot trials, future confirmatory studies should consider enrolling approximately 25–30 participants per group to ensure robust evaluation. Further research should also explore whether treatment response to VCT varies according to disease severity (e.g., OA grade), body mass index, age, or other clinical characteristics, as suggested in other rehabilitation studies [65,66,67,68,69,70,71]. Moreover, systematic investigation of different pressure protocols, including variations in positive and negative pressure ratios and cycle duration, may help identify optimal therapeutic parameters. Such analyses could ultimately support more individualized treatment strategies tailored to specific patient profiles. The use of objective methods, such as Doppler ultrasound, infrared thermography, or biochemical markers, could strengthen the robustness of future studies by enabling more precise characterization of tissue perfusion and providing greater insight into the underlying mechanisms of VCT.

## 5. Conclusions

In this pilot comparative study, the addition of VCT to standard physical therapy was associated with greater improvement in functional outcomes in patients with knee OA. While pain intensity or mental health outcomes were comparable between groups, clinically meaningful gains in physical function were more frequently observed in the intervention group. Taken together, the observed results point toward a possible adjunctive role of VCT in improving functional outcomes in patients with knee OA. Given the exploratory design of the study, these findings should be viewed as preliminary and interpreted with caution. Rather than supporting immediate clinical implementation, they provide an initial basis for the design of future randomized controlled trials. Confirmation in larger, adequately powered studies is required.

## Figures and Tables

**Table 1:** Baseline characteristics of participants.

Variable		Control Group (n = 16)	Intervention Group (n = 16)
Age, years (mean ± SD)		67.9 ± 7.8	66.0 ± 6.6
Female sex, n (%)		12.0 (75%)	14.0 (88%)
KL grade, n (%)	Grade 1	3.0 (19%)	1.0 (6%)
	Grade 2	3.0 (19%)	4.0 (25%)
	Grade 3	10.0 (63%)	11.0 (69%)
BMI category, n (%)	Normal weight	6.0 (38%)	8.0 (50%)
	Overweight	7.0 (44%)	3.0 (19%)
	Obese	3.0 (19%)	5.0 (31%)
WOMAC, median (IQR)		49.0 (39.5–57.3)	46.5 (38.8–57.8)
VAS, median (IQR)		7.0 (5.8–8.0)	7.0 (5.8–8.0)
PCS, median (IQR)		34.0 (31.5–38.0)	36.0 (32.8–37.5)
MCS, median (IQR)		37.0 (35.0–39.3)	41.5 (39.0–45.5)

Abbreviations: IQR, Interquartile range; KL grade, Kellgren-Lawrence grade; MCS, mental component of the SF-12; PCS, physical component of the SF-12; SD, standard deviation; VAS, visual analog scale; WOMAC, Western Ontario and McMaster Universities Osteoarthritis Index.

**Table 2:** Within-group changes in outcome measures from baseline to 1-month follow-up.

	Control Group			Intervention Group		
Outcome	1-Month Follow-Up	Δ (%)	*p*	1-Month Follow-Up	Δ (%)	*p*
WOMAC	41.0 (31.5–50.0)	18% (12–28%)	<0.001	25.0 (19.3–40.0)	65% (18–118%)	<0.001
VAS	3.0 (2.0–4.0)	54% (47–61%)	<0.001	2.5 (1.0–4.0)	65% (39–78%)	<0.001
PCS	35.5 (33.5–40.0)	5% (0–10%)	0.008	43.0 (37.3–47.25)	22% (8–40%)	<0.001
MCS	37.5 (37.0–45.5)	3% (0–18%)	0.014	44.0 (40.0–52.5)	3% (0–18%)	0.008

Data are presented as median (interquartile range), and Δ (%) represents median percentage change from baseline. Within-group comparisons were evaluated using the Wilcoxon signed-rank test, and results were considered statistically significant at *p* < 0.05. Abbreviations: MCS, mental component of the SF-12; PCS, physical component of the SF-12; VAS, visual analog scale; WOMAC, Western Ontario and McMaster Universities Osteoarthritis Index.

**Table 3:** Between-group comparison of outcome measures at 1-month follow-up adjusted for baseline values (ANCOVA).

	Adjusted Mean Difference (95% CI)	*p*	Partial eta^2^
WOMAC	−9.87 (−16.30 to −3.43)	0.004	0.25
VAS	−0.39 (−1.42 to 0.64)	0.4	0.02
PCS	6.39 (2.99 to 9.80)	<0.001	0.34
MCS	2.23 (−1.14 to 5.60)	0.186	0.06

Abbreviations: MCS, mental component of the SF-12; PCS, physical component of the SF-12; VAS, Visual Analog Scale; WOMAC, Western Ontario and McMaster Universities Osteoarthritis Index.

**Table 4:** MCID responder analysis at 1-month follow-up.

Outcome	MCID Threshold	Control	Intervention	Risk Difference (95% CI)	Risk Ratio (95% CI)
WOMAC	≥12 points	25%	62.5%	0.38 (0.06 to 0.69)	2.50 (1.00 to 6.23)
VAS	≥2 points	100%	100%	0	Not estimable
PCS	≥5 points	18.8%	68.8%	0.50 (0.20 to 0.80)	3.67 (1.29 to 10.40)
MCS	≥5 points	31.3%	43.8%	0.13 (−0.21 to 0.46)	1.40 (0.53 to 3.71)

Responders were defined as participants achieving the predefined MCID threshold for each outcome. Risk difference and risk ratio are presented with 95% confidence intervals. Abbreviations: MCID, minimal clinically important difference; MCS, mental component of the SF-12; PCS, physical component of the SF-12; VAS, Visual Analog Scale; WOMAC, Western Ontario and McMaster Universities Osteoarthritis Index.

## Data Availability

The data presented in this study are available on request from the corresponding author. The data are not publicly available due to privacy and ethical restrictions.

## Abbreviations

The following abbreviations are used in this manuscript:

OA	Osteoarthritis
TKA	Total knee arthroplasty
KL grade	Kellgren–Lawrence radiographic grading scale for osteoarthritis severity
VCT	Vacuum-compression therapy
WOMAC	Western Ontario and McMaster Universities Osteoarthritis Index
VAS	Visual Analog Scale
SF-12	12-Item Short Form Health Survey
PCS	Physical Component Summary (subscore of the SF-12 questionnaire)
MCS	Mental Component Summary (subscore of the SF-12 questionnaire)
MCID	Minimal Clinically Important Difference
IQR	Interquartile Range
SD	Standard Deviation
CI	Confidence Interval
ANCOVA	Analysis of Covariance
